# Incorporating Irrigation Channels in Sleeved Surgical Guides

**DOI:** 10.7759/cureus.82460

**Published:** 2025-04-17

**Authors:** Todor Uzunov, Alex Yankov, Raya G Grozdanova, Todor G Bogdanov

**Affiliations:** 1 Department of Prosthetic Dental Medicine, Faculty of Dental Medicine, Medical University of Sofia, Sofia, BGR; 2 Department of Prosthodontics, Faculty of Dental Medicine, Medical University of Sofia, Sofia, BGR; 3 Department of Conservative Dentistry, Faculty of Dental Medicine, Medical University of Sofia, Sofia, BGR; 4 Department of Medical Physics and Biophysics, Faculty of Medicine, Medical University of Sofia, Sofia, BGR

**Keywords:** 3d printing, dental implants, irrigation channels, osteotomy, surgical drilling, surgical guide

## Abstract

A critical stage in dental implant procedures involves preparing the implant site via surgical drilling. Adequate irrigation during this process is crucial to prevent thermal damage to the surrounding bone tissue. While surgical guides significantly improve the accuracy of implant site preparation by directing drill movement, they can also obscure the operative field and impede effective coolant delivery.

This report presents design modifications to surgical guides compatible with the Straumann® system (Institut Straumann AG, Switzerland) to enhance coolant access and optimize temperature control during drilling.

Three types of surgical guides were developed using specialized computer-aided design software. The first design is a conventional guide without irrigation channels. The second incorporates a single irrigation channel, while the third-an innovative prototype-features a dual-channel irrigation system. In both modified designs, the irrigation outlets are strategically positioned below the lower edge of the guiding sleeve to improve coolant delivery to the drilling site.

The results confirm the feasibility of producing this new dual-channel surgical guide. Implantology professionals can replicate and evaluate this design by following the outlined modifications, thereby enhancing both safety and efficacy in guided implant surgery.

## Introduction

In modern dental implantology and oral surgery, technology plays a key role in ensuring high precision in dental implant placement, bone augmentation, and other surgical procedures. It enhances the accuracy, safety, and efficiency of surgical interventions by guiding surgical instruments such as drills and blades to precisely determined positions, minimizing errors, and improving outcomes. In dental practice, three types of surgical guides are commonly used: 1) diagnostic guides that are used for planning and visualizing the final position of prosthetic constructions, 2) navigational guides that direct instruments during surgical intervention, and 3) implantological guides that are specifically designed for the precise positioning of dental implants.

Nowadays, these guides are most commonly designed using computer-aided design (CAD)/computer-aided manufacturing (CAM) technologies [1], utilizing 3D models created through cone beam computed tomography (CBCT) and intraoral scanning. They are fabricated from biocompatible materials, such as transparent acrylic resins or medical-grade polymers, ensuring high accuracy and sterility. These guides reduce the risk of complications, improve the precision of surgical procedures, shorten the duration of operations, and enhance postoperative patient recovery.

One of the most significant technological breakthroughs in this field is using surgical guides, which provide precise navigation for dental implant placement. This report explores the development of an innovative implantological guide, which will be manufactured using 3D printing technology with Dental Yellow Clear Resin from HARZ Labs (Podgorica, Crna Gora).

The key innovation in this project is the integration of water-cooling channels into the structure of the surgical guide. This novel approach aims to minimize thermal stress on bone tissue during osteotomy, thereby reducing the risk of necrosis and accelerating postoperative patient recovery. Including water-cooling channels represents a significant advancement in implantological surgical guides, offering better protection for biological structures and increasing the efficiency of surgical interventions.

The report will detail the entire process of designing and manufacturing this innovative surgical guide, including an analysis of the CAD/CAM technologies used, the characteristics of the Dental Yellow Clear Resin photopolymer material, and the expected clinical benefits of the innovation. Furthermore, it will discuss challenges in integrating cooling channels and their potential applications in future dental surgical practices.

Following the emergence of the first studies [2] in the early 21st century, technological advancements have led to the continuous refinement of this technology. In one such study, the authors presented a case of computer-guided implantology, where a surgical guide was used for flapless surgery with immediate loading. This research demonstrated the potential of computer-assisted planning and the use of surgical guides in improving accuracy and predictability in dental implant placement.

Since the introduction of computer-guided implantology procedures in the early 21st century, technology in this field has advanced rapidly. Today, 3D-printed surgical guides are widely used in clinical practice [3], offering high precision and improved treatment outcomes. However, one of the key challenges during osteotomy remains the risk of thermal damage to the bone caused by temperature elevation during drilling. As early as the 1950s, this issue was recognized, with research indicating that exposure to temperatures above 47°C for one minute can result in localized bone resorption. Such thermal exposure leads to microvascular dilation lasting several days and damage to adipose tissue in the affected area [4]. The extent of temperature rise during drilling is influenced by multiple factors, including the characteristics of the instruments used, the drilling technique, the applied force, the efficiency of the cooling method, the wear and design of the drills [5], and the bone density at the implant site [6].

Bone cooling during surgical drilling is most commonly achieved by directing a stream of physiological saline toward the operative area. For effective cooling, the fluid must reach the tip of the rotating instrument. Standard surgical guides often limit both visual and physical access to the working field, thereby interfering with the proper delivery of the coolant. As a result, they can lead to a significantly higher temperature increase compared to freehand drilling techniques. These limitations of conventional surgical guides can lead to clinically significant complications, such as delayed osseointegration, increased postoperative discomfort, and, in severe cases, partial implant failure due to thermally induced bone necrosis. In response to these concerns, several modified guide designs have been introduced in clinical and experimental settings. For example, some 3D-printed guides incorporate internal irrigation channels to allow more direct coolant delivery to the osteotomy site, mitigating thermal risks. Others include dual-channel systems or auxiliary tubes modeled virtually to align with the drilling trajectory, aiming to optimize coolant distribution without compromising guide stability. While these innovations show promise, many remain limited to experimental setups or require complex customization, highlighting the need for practical, adaptable solutions suitable for routine clinical use.

Despite this limitation, surgical guides remain essential tools for achieving optimal implant positioning and improved procedural accuracy. Multiple solutions have recently emerged to improve cooling efficiency during implant site preparation. For instance, certain 3D-printed guides feature built-in channels or tubes designed to deliver coolant directly to the osteotomy site. In some designs, the irrigation tube is modeled as a virtual custom implant, aligned along the desired fluid direction [7]. Other studies, such as those by Teich et al., have demonstrated that internal irrigation channels significantly improve cooling efficiency [8]. Similarly, Parvizi et al. developed a guide with both inlet and outlet channels, reporting a notable reduction in intraosseous temperature in experimental settings [9].

The primary objective of the present study was to develop an in-house workflow for designing and 3D printing surgical guides with integrated cooling features, using standard implant planning software. This method aims to allow for anatomical customization and easy adaptation to different clinical cases. The study focuses on evaluating the thermal performance of the proposed design compared to conventional cylindrical and open-sleeve surgical guides.

## Technical report

Creating and validating a surgical guide model can be divided into several key steps. These include gathering information through CBCT and intraoral scanning, creating a digital model of the dental arch, developing a computer model of the surgical guide using specialized software, fabricating three-dimensional (3D) physical models using 3D printing technology, and validating its applicability.

A preplanning CBCT scan was obtained for all samples. A digital impression was also taken for all samples using an intraoral scanner (Trios 5, 3Shape A/S, Copenhagen, Denmark). The surface files were exported as Standard Tessellation Language (STL). Then, the intraoral scan was used to create the model of the teeth.

**Figure 1:**
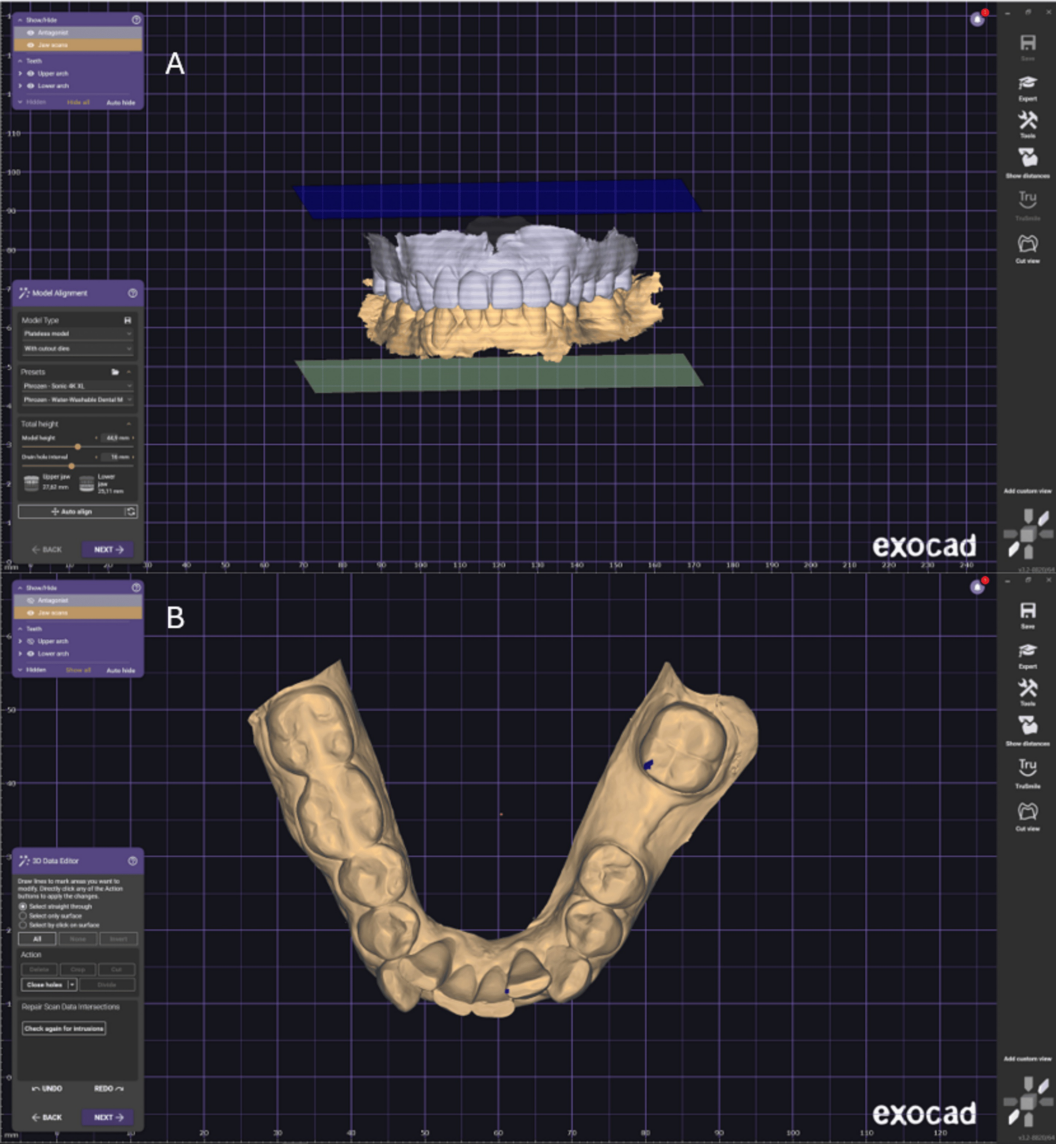
Development of the dental model in Exocad using Model Creator. (A) Scanned bite in occlusion. (B) Isolated lower jaw, representing the area of interest

The model was developed in Exocad (Exocad GmbH, Darmstadt, Germany) using Model Creator. The model type used in Model Creator was a plateless model. The excess of the intraoral scan was removed to obtain a clean model (Figure 1A). To create the solid model, the “Hollow Model” option in the Settings tab was unchecked, and the model was generated (Figure 1B).

**Figure 2:**
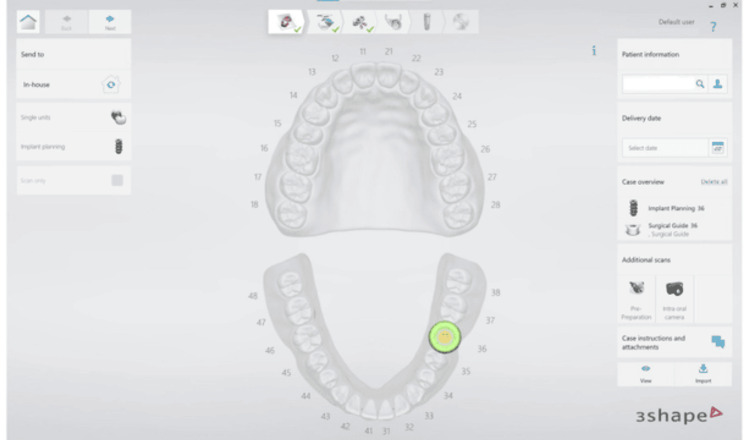
Importing CBCT and STL files into 3Shape Implant Studio for implant planning and surgical guide design CBCT: cone beam computed tomography; STL: Standard Tessellation Language

For the surgical guide, the CBCT and STL files were imported into the 3Shape Implant Studio Software (3Shape A/S, Copenhagen, Denmark). In the implant planning phase, the lower jaw was selected, and the Guide Workflow was tooth-supported. Tooth 36 was chosen as the implant site (Figure 2).

**Figure 3:**
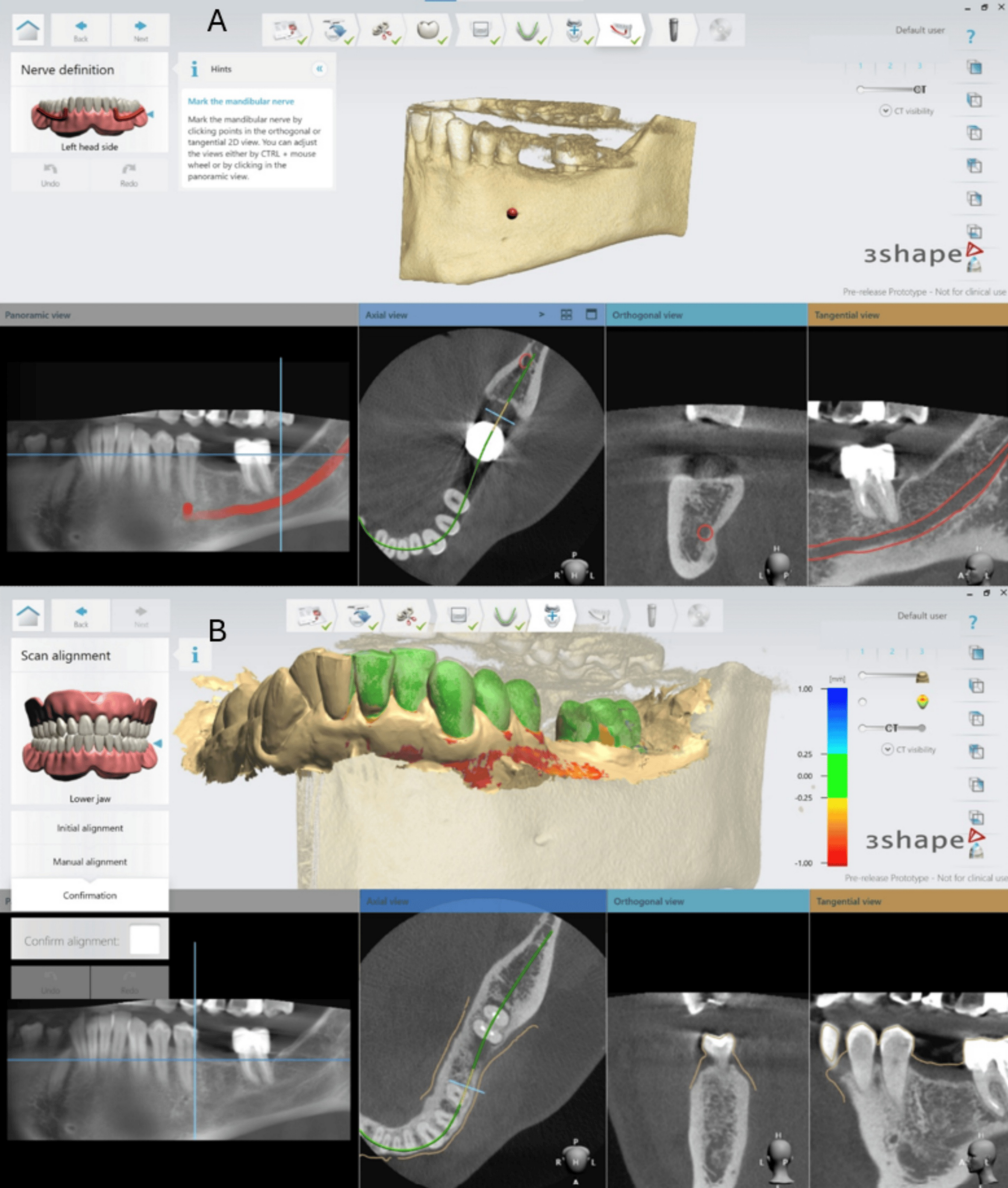
CBCT scan processing: panoramic curve adjustment, alignment of intraoral and CBCT scans (A), and marking of the mandibular nerve (B) CBCT: cone beam computed tomography

The CBCT scan was cropped, and the panoramic curve was drawn. The intraoral and CBCT scans were aligned to ensure a proper match (Figure 3A). The mandibular nerve was marked (Figure 3B).

**Figure 4:**
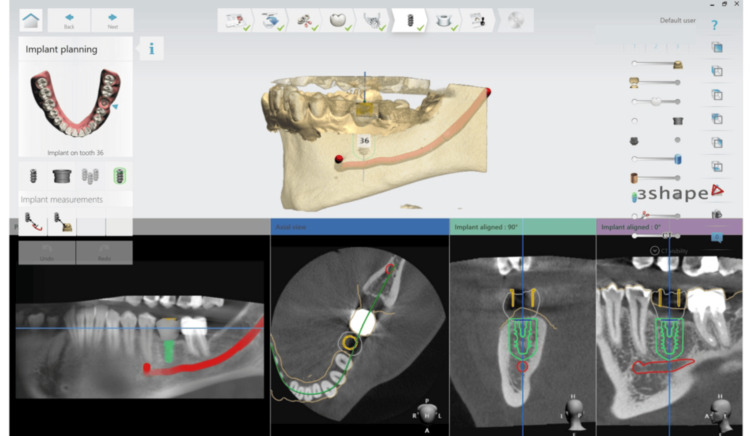
Implant and sleeve selection: BLX Roxolid SLActive implant (5 mm × 10 mm) and T-sleeve PEEK guide by Straumann

We used BLX Roxolid SLActive by Straumann (Straumann, Basel, Switzerland), which has a diameter of 5 mm and a length of 10 mm. For the sleeve, we utilized the T-sleeve PEEK by Straumann as a guide for the implant drill. The diameter of the sleeve was also 5 mm (Figure 4).

Once the implant planning was complete, we proceeded with the design of the guide. The guide border was drawn, a support bar was added, and windows were designed. After that, the guide was exported as an STL file.

**Figure 5:**
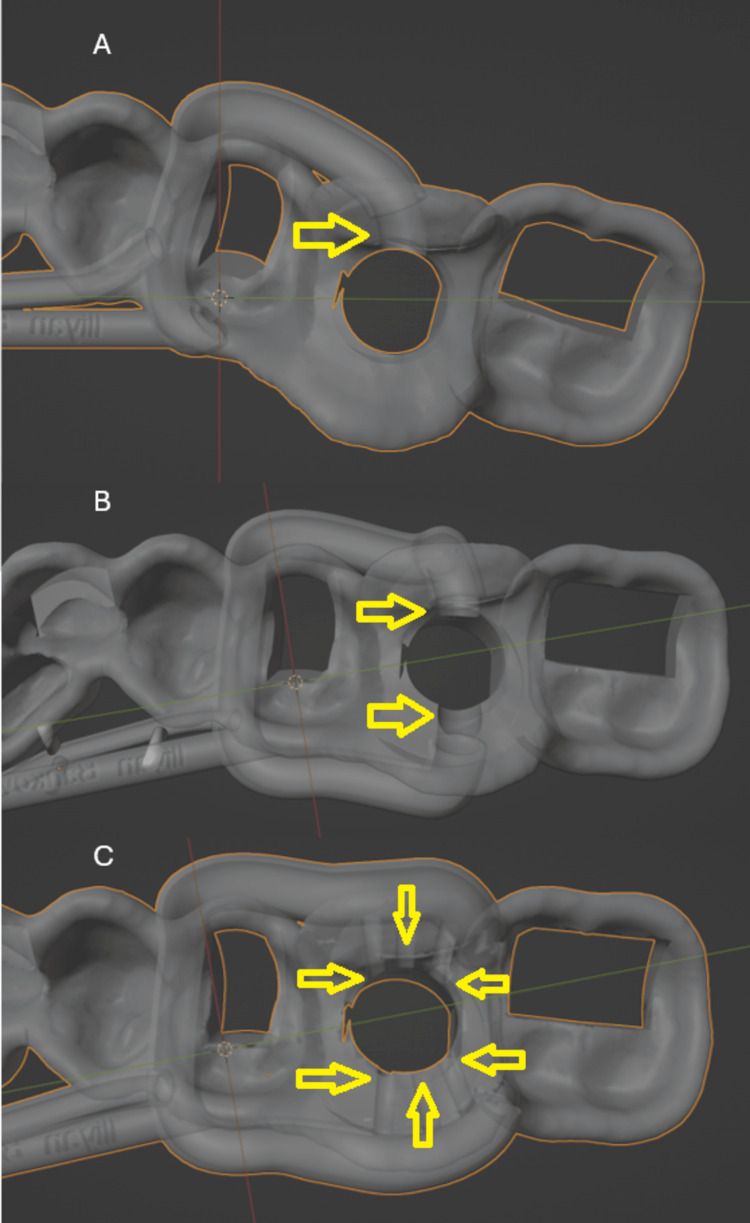
Visual comparison of surgical guide designs with integrated irrigation channels, demonstrating progression from a single-channel system (A) to multiple-channel configurations (C), enhancing coolant distribution from two-channel configuration (B)

We designed three variations of irrigation-enhanced guides to explore the most effective approach for coolant delivery. The single-channel guide serves as an initial modification to improve coolant access in a minimally invasive manner, appropriate for short osteotomy procedures with limited thermal risk. The dual-channel design with two outlets provides bilateral irrigation and is indicated for standard-length drilling in denser bone, where greater coolant coverage is needed. Finally, the dual-channel design with multiple outlets is intended for complex or prolonged procedures, where uniform coolant distribution along the entire osteotomy path is essential. These design iterations allowed us to compare structural complexity vs. potential cooling benefit and to establish a scalable concept adaptable to diverse clinical needs. For the creation of the irrigation channels, the STL file of the guide was imported into Blender (Blender Foundation, The Netherlands). The creation of the channels began with adding a Bezier curve. From there, in the “Data” tab under “Geometry,” the diameter of the channel was entered. The main channel has a diameter of 2.5 mm. The extra channels have a diameter of 1-1.5 mm. The curve was then converted into a mesh. Using the “Boolean” modifier, the exit holes were generated, as shown in Figure 5.

Once the guide with the irrigation channels was created, it was exported as an STL file. After the model and the guides were created, they were 3D-printed. Fused deposition modeling technology was used to create the dental arch model. The printing was performed on a Bambu Lab X1C (Bambu Lab, Austin, TX) using standard polylactic acid filament 3DLine (MONICONI OOD, Yambol, Bulgaria). Standard printing settings were applied, with a layer thickness of 0.2 mm and a nozzle diameter of 0.4 mm. The sequence of designs in Figure 5 highlights the increasing complexity and coverage of irrigation, offering enhanced cooling potential with each iteration.

The fabrication of the various surgical guide variations was carried out using a Phrozen XL 4K Plus (Phrozen Tech Co., Ltd., Hsinchu City, Taiwan) with Dental Yellow Clear Resin. The printing settings were as follows: layer thickness: 50 µm, retraction distance: 7.00 mm (for the base: 8.00 mm); number of base layers: 6; base curing time: 40.00 seconds; base lift height: 8.00 mm; normal curing time: 4.00 seconds; base and normal peel speeds: 50.00 mm/minute; base and normal return speeds: 100.00 mm/minute.

**Figure 6:**
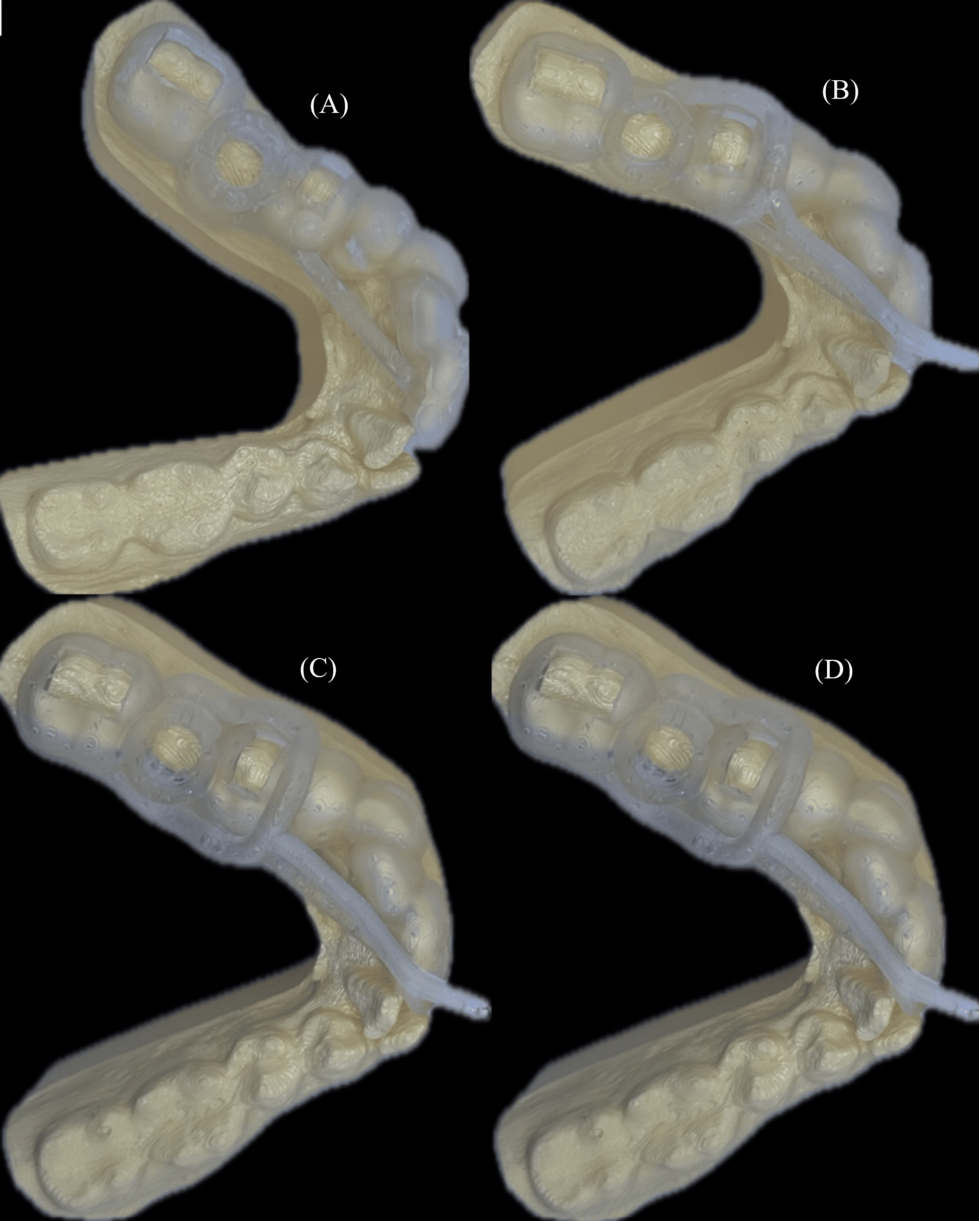
3D printed dental arch and variations of the surgical guides, illustrating proper model alignment and guide adaptation to anatomical structures. (A) Surgical guide without cooling channel. (B) Guide with a single cooling channel. (C) Guide with two cooling channels. (D) Guide with multiple cooling channels 3D: three-dimensional

Figure 6 presents the results of the printed dental arch and the different variations of the surgical implant guides, along with the alignment of the models.

**Figure 7:**
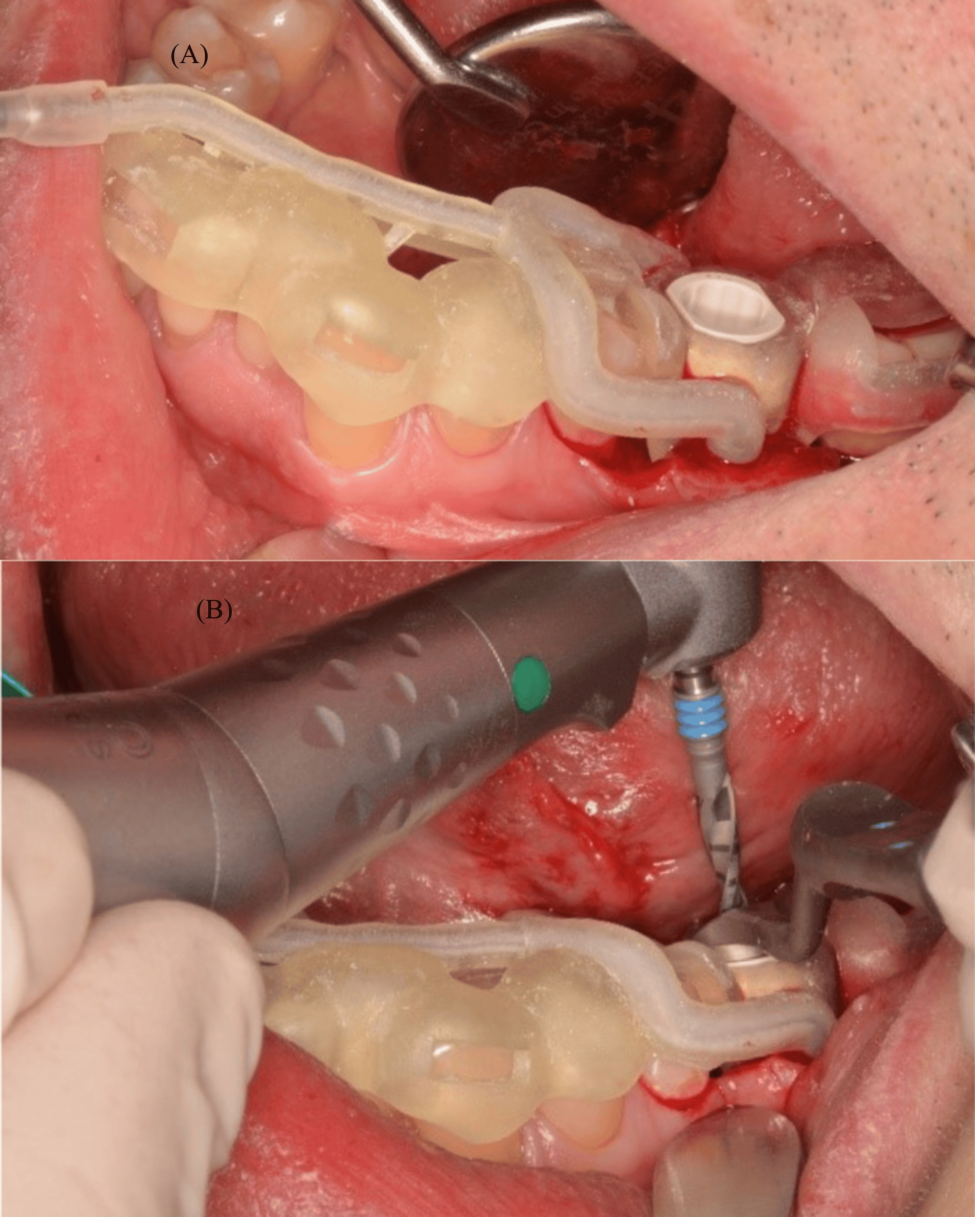
The intraoperative use of the dual-channel guide, showing its integration (A) and practical application (B) into the surgical workflow

The fabricated surgical guides were used to perform an implantology procedure after placing the corresponding sleeve. During the procedure, the variation with two irrigation channels was utilized. Figure 7 illustrates the use of the guide during the procedure. The dual-channel guide was applied during a demonstration procedure to illustrate the positioning and coolant flow in a realistic clinical setting. This case was chosen for visualization purposes only and not based on specific inclusion criteria. The guide variations were developed progressively, with each iteration aiming to improve irrigation efficiency in response to the well-documented risk of thermal damage during osteotomy, as discussed in the Introduction section. This figure also illustrates the alignment between the guide and the dental arch, confirming its suitability for intraoral use. Although not shown in multiple steps, the guide was used throughout the drilling process, with irrigation delivered consistently through the integrated channels.

Although no intraoperative temperature measurements were conducted in this study, an irrigation system was employed during all drilling steps. Sterile saline was delivered continuously using external tubing connected to a standard dental unit at a flow rate of approximately 40 mL/minute. An integrated irrigation line connected directly to the surgical handpiece was not used. Instead, external tubing was positioned to ensure direct and continuous application of coolant to the osteotomy site, facilitating effective heat management. The final surgical guide utilized featured a dual-channel system with multiple outlets, designed specifically to maximize fluid distribution across the drilling area.

## Discussion

While the figures primarily depict the design and application phases, the integrated irrigation system was actively used during all steps of osteotomy. The coolant was delivered continuously, minimizing thermal accumulation throughout the procedure. The present study introduces a significant innovation in the design of surgical guides by integrating cooling channels below the lower edge of the guiding sleeve. This structural improvement eliminates a major limitation of conventional surgical guides, namely the restricted access of the cooling fluid to the drilling site. In traditional surgical guides, the sleeve obstructs the flow of physiological saline, leading to a substantial increase in bone temperature and elevating the risk of thermal damage. With the new design, the cooling fluid is directed precisely to the critical area, ensuring efficient heat dissipation and preventing excessive bone heating [8].

Establishing uniform and direct cooling at the drilling site is essential for reducing the risk of bone necrosis. Studies by Eriksson and Albrektsson have demonstrated that exposure to temperatures above 47°C for more than one minute can result in irreversible bone tissue damage [4]. There is a high risk of exceeding this thermal threshold in standard surgical guides, where cooling does not effectively reach the osteotomy site. In the newly designed guide, the cooling fluid is evenly distributed across the entire osteotomy area, allowing for better thermal gradient management and maintaining bone tissue within safe temperature limits.

Compared to standard external cooling, which relies on a directed stream of physiological saline, the new design exhibits significant advantages. Traditional guides limit the effectiveness of cooling fluid as the sleeve prevents it from adequately reaching the drilling site. This leads to uneven cooling and creates hot spots in the bone structure, potentially compromising implant osseointegration [5]. The internal cooling channels implemented in this study are positioned below the lower edge of the guiding sleeve, ensuring a continuous and direct flow of cooling fluid toward the active surgical area. This modification builds upon previous research, such as the findings of Teich et al., which demonstrated that using internal cooling channels significantly reduces thermal stress on bone tissue [8].

Another key advantage of the new design is its flexibility and adaptability to different implant systems. While the present study is based on the Straumann system, the concept of cooling channels can be applied to other platforms by adjusting the sleeve diameter and the positioning of the cooling channels. Incorporating these structural enhancements does not require significant modifications to the standard CAD/CAM software used for surgical guide design. This ensures broad applicability of the proposed methodology, making it suitable for integration into various dental practices [7].

Despite the advantages of the innovative cooling mechanism, certain potential challenges should be considered. The inclusion of multiple cooling channels may increase aerosol formation, which poses a risk for microorganism dispersion within the surgical field. This is particularly critical in the context of infection control and surgical sterility. Future developments could focus on strategies for minimizing aerosol effects, such as optimizing the diameter of the cooling channels or controlling the flow rate of the cooling fluid [9].

Further refinement of the method could involve the development of dynamic cooling systems, which adjust the fluid flow according to drilling depth and bone characteristics. A regulated flow rate would allow for customized cooling based on specific clinical conditions, contributing to greater efficiency and safer surgical procedures.

Another potential aspect of future advancements is using antibacterial coatings on surgical guides. Incorporating silver nanoparticles or antibacterial photopolymers in the production of guides could significantly reduce the risk of infection, especially in prolonged procedures. The combination of innovations in cooling mechanisms and antimicrobial technologies could lead to substantial improvements in clinical outcomes and patient safety [10].

The various guide designs presented in this study address common limitations of conventional systems by enhancing coolant access. Clinical indications for the dual-channel designs include implant placements in posterior regions with reduced visibility and access, patients with dense bone, or longer drilling sequences. The final prototype, with dual channels and multiple outlets, offers improved fluid dispersion across the osteotomy site, potentially lowering peak temperatures during drilling. Although this report focuses on design and feasibility, future studies will incorporate quantitative validation, including intraosseous temperature measurements.

Table 1 presents a comparative summary of the four guide designs tested in this study. Each variation introduces different levels of irrigation enhancement, offering progressively improved coolant distribution and surgical precision. The final prototype, with dual channels and multiple outlets, demonstrates the highest potential for clinical performance due to its ability to deliver irrigation fluid broadly and efficiently to the osteotomy site.

**Table 1:** Comparison of surgical guide designs

Surgical guide design	Irrigation type	Key feature	Intended advantage
Conventional	External only	No internal channels	Limited coolant access; risk of overheating
Single channel	Internal + external	One internal channel, single outlet	Improved but localized coolant delivery
Dual channel (two outlets)	Internal + external	Two channels, one outlet per channel	Bilateral fluid entry; better cooling coverage
Dual channel (multiple outlets)	Internal + external	Two channels with multiple outlet ports	Maximum coolant dispersion; optimal thermal regulation

## Conclusions

The innovative surgical guide design presented in this study, featuring cooling channels positioned below the lower edge of the guiding sleeve, offers significant advantages over traditional guides. It ensures improved cooling, a reduced risk of bone necrosis, and greater accuracy in implant placement. Additionally, its versatility and compatibility with various implant systems make it a promising technology for future applications. Future research may focus on regulating the cooling fluid flow rate, minimizing aerosol effects, and integrating antimicrobial properties to further enhance the clinical performance of surgical guides.
